# Fear of Falling and Cognitive Decline in Healthy Aging Adults

**DOI:** 10.1093/geroni/igaa057.849

**Published:** 2020-12-16

**Authors:** Rebecca Kraut, Roee Holtzer

**Affiliations:** 1 Yeshiva University, New York, New York, United States; 2 Yeshiva University, Bronx, New York, United States

## Abstract

Fear of Falling (FOF) is common and associated with poor mobility in aging but whether persistence of FOF endorsement influences cognitive decline has not been reported. Here we determined the effect of FOF, measured dichotomously and after accounting for persistence, on decline in global cognitive function (GCF), memory, and attention/executive functions. Older adults with persistent FOF (n=81; mean age=77.63±6.67 yrs; %female=74.1), transient FOF (n=60; mean age=76.93±6.01 yrs; %female=61.7), and no FOF (n=286; mean age=75.77±6.42 yrs; %female=49.3) were included. FOF was assessed through yes/no responses to “do you have a fear of falling?” at baseline. GCF was assessed using RBANS; memory was assessed using a composite score comprising the immediate and delayed recall index scores from RBANS; attention/executive functions were assessed via a composite score comprising TMT A & B, letter and category fluency tasks, and digit symbol modalities. Cognitive measures were administered annually for up to six years. Linear mixed effects models revealed that persistent FOF was associated with a worse decline in GCF compared to both transient FOF (estimate=0.78, p=.022) and no FOF (estimate=0.75, p=.004). Persistent FOF was also associated with a worse decline in memory compared to those with transient FOF (estimate=0.08, p=.004) and those with no FOF (estimate=0.06, p=.006). Associations between FOF status and decline in attention/executive functions were not significant. These findings demonstrate that persistent FOF is a risk factor for cognitive decline in community-residing older adults.

